# Parent, child, and environmental predictors of vegetable consumption in Italian, Polish, and British preschoolers

**DOI:** 10.3389/fnut.2022.958245

**Published:** 2022-10-21

**Authors:** Natalie A. Masento, Katrina May Dulay, Kate Harvey, Daniela Bulgarelli, Marcella Caputi, Giuseppina Cerrato, Paola Molina, Katarzyna Wojtkowska, Dominika Pruszczak, Julia Barlińska, David Messer, Carmel Houston-Price

**Affiliations:** ^1^School of Psychology & Clinical Language Sciences, University of Reading, Reading, United Kingdom; ^2^Department of Psychology, City, University of London, London, United Kingdom; ^3^Department of Psychology, University of Turin, Turin, Italy; ^4^Department of Life Sciences, University of Trieste, Trieste, Italy; ^5^Department of Chemistry, University of Turin, Turin, Italy; ^6^Department of Regional & Urban Studies and Planning, University of Turin, Turin, Italy; ^7^Faculty of Psychology, University of Warsaw, Warsaw, Poland; ^8^Faculty of Wellbeing, Education & Language Studies, Open University, Milton Keynes, United Kingdom

**Keywords:** vegetable intake, cross-cultural, family environment, eating behaviour, parenting style, child temperament

## Abstract

This study compared the vegetable intake of preschool children from three European countries [Italy, Poland, and the United Kingdom (UK)] and explored the parent, child, and environmental factors that predicted intake in each country. A total of 408 parents of preschoolers (Italy: *N* = 61, Poland: *N* = 124, and UK: *N* = 225; child mean age = 32.2 months, *SD* = 9.47) completed an online survey comprising a set of standardised questionnaires. For all three countries, the questionnaires included measures of children’s vegetable intake (VegFFQ), child eating behaviour (CEBQ-FF), parents’ mealtime goals (FMGs), and sociodemographic questions about family background and environment. In the UK and Italy, additional questionnaires were used to assess child temperament (EAS-T) and parents’ feeding practices (CFPQ). The results showed that the number of child-sized portions of vegetables consumed per day varied significantly across countries; Polish children consumed the most (∼3 portions) and Italian children the least (∼1.5 portions). Between-country differences were seen in parents’ goals for family mealtimes; compared to Italian parents, Polish and UK parents were more motivated to minimise mealtime stress, increase family involvement in meal preparation, and share the same foods with family members. British and Italian parents also adopted different feeding practices; parents in the UK reported more use of healthy modelling behaviours and more use of foods to support their child’s emotion regulation. In terms of child factors, Italian children were reported to be more emotional and more sociable than British children. Analyses of the relationships between the parent, child, and environmental factors and children’s vegetable intake revealed both similarities and differences between countries. Negative predictors of vegetable intake included child food fussiness in the UK and Poland, child temperament (especially, shyness) in Italy, and the use of food as a reward and child emotionality in the UK. Positive predictors included the parental mealtime goal of ‘family involvement’ in the UK. These results highlight differences in the extent to which European preschoolers achieve recommended levels of vegetable intake, and in the factors that influence whether they do. The results suggest a need to develop healthy eating interventions that are adopted to meet the specific needs of the countries in which they are implemented.

## Introduction

Consumption of a varied and vegetable-rich diet in early childhood predicts lifelong dietary variety and good health (1–3). Although evidence for a direct link between fruit and vegetable intake and obesity is more mixed for children than for adults (4, 5), examination of children’s diets highlights that low fruit and vegetable consumption tends to be coupled with high fat and sugar consumption (6), which increases the risk of obesity and overweight (7, 8). Reports on steadily increasing rates of overweight and obesity in young children (9) highlight the need for obesity prevention initiatives that are evidence-based and informed by the latest understanding of factors that support healthy food choices, including greater vegetable intake.

This global health landscape is mirrored in the quality of children’s diets in Europe, where many countries report a high prevalence of overweight or obese children. However, the picture is not uniform. One assessment conducted between 2011 and 2016 (10) compared the proportion of 2- to 7-year-olds who were overweight or obese in different regions of Europe to comparable statistics captured between 1999 and 2006. While Central Europe (including Poland) saw a decline in the proportion of overweight or obese children during this period (from 16 to 13%), Mediterranean regions (including Italy) saw a small increase in prevalence (from 19 to 20%). Although data were not collected in the Atlantic region (including the UK) between 2011 and 2016, this region showed a relatively stable prevalence of around 12% between 1999 and 2010 (10). These figures show that overweight and obesity are significant issues for children across Europe, and that prevalence patterns are not uniform and may not align with commonly held stereotypes of dietary quality in different countries.

Further evidence that children across Europe are failing to achieve healthy diets is seen in reported levels of fruit and vegetable consumption (11, 12). Recommended levels of fruit and vegetables intake are highly consistent across countries; the WHO recommends that individuals eat at least 400 g of fruit or vegetable per day to support good health (13), and public health guidelines across Europe promote the consumption of a standard five portions per day, roughly equivalent to the WHO guidance [the UK (14), Italy (15), and Poland (16)]. Between 2015 and 2017, the WHO Childhood Obesity Surveillance Initiative (COSI) (11) collected data in 23 countries (including Italy and Poland) about the fruit, vegetable, and snack intake of 6- to 9-year-old children. The results revealed that only 23% of the children sampled ate vegetables every day and that rates varied widely between countries (54% in Italy and 23% in Poland). Other assessments of children’s diets in Poland (17) and Mediterranean regions (18) provide a less pessimistic view. In the UK, the Health Survey England data show that only 18% of children aged 5–15 years eat the recommended five portions of fruit and vegetables per day, while a similar proportion typically eats none at all (12). While there are inconsistencies in the literature about the precise level of children’s fruit and vegetable intake, these reports suggest that there are considerable differences in these levels across European countries.

The causes of these differences, in both overweight and obesity rates and in levels of fruit and vegetable consumption, are complex and likely to include environmental, family, parent, and individual child factors. Given that food preferences and eating behaviours become established in early childhood (19) and track from childhood to adulthood (20), a better understanding of how these factors are involved in the formation of food preferences and dietary outcomes is essential to efforts to develop effective public health initiatives. This article is the first to investigate how environmental, parent, and individual child factors contribute to children’s vegetable intake and whether these relationships are stable across countries or specific to particular populations.

In terms of the influence of the eating environment, European countries show a broad diversity in a range of cultural, demographic, and socioeconomic factors likely to impact public health. Cultural differences include the extent to which different populations have adopted the widespread shift from a traditional Mediterranean diet, which relies heavily on fruits and vegetables, to a more Western diet that includes more foods high in sugar, salt, and fat (10, 18). The socioeconomic status of different populations is also an important consideration given the links between economic hardship and the ability to achieve a healthy balanced diet, and we note in particular the increased prevalence of poverty, health inequality, and unemployment in Southern Europe (including Italy) following the financial crisis of 2007 (10, 21, 22). Research suggests that some socioeconomic and demographic factors such as maternal level of education and socioeconomic status are associated with children’s fruit and vegetable consumption, but that other demographic factors including ethnicity are not (23).

Within the home environment, parents (and other caregivers) play a key role in shaping children’s food preferences and eating behaviours (19, 24–26). Parents are the primary gatekeepers to the foods children eat, controlling the availability and accessibility of vegetables at home, which are strong predictors of preschool children’s willingness to eat vegetables (27, 28). Parents also shape the environment in which foods are eaten. Mealtimes provide the opportunity to expose children to a variety of foods and for children to eat these with parents and caregivers who model positive feeding practices (29–31). Parental modelling of food acceptance plays a powerful role in how children learn about and engage with food (32–35), such that parents’ dietary preferences very often predict those of their children (36–39). Parents’ feeding styles, practices, and emotional responses are also known to influence a child’s relationship with food (24, 40, 41). Positive feeding practices such as reasoning, praise, and encouragement are associated with greater vegetable consumption by children (42, 43), while negative feeding practices such as pressure to eat, negative comments, and food restriction are linked to lower vegetable intake (44). Parental feeding practices are related to their broader parenting style but can be modified by appropriate education and support; tailored public health initiatives that target parent feeding styles have met with some success (45). Finally, children’s eating behaviour is also linked to the extent to which parents hold positive mealtime goals (31); those known to support healthy eating include the goal to reduce stress and conflict around mealtimes, the goal to provide positive modelling opportunities by sharing the same foods, and the goal to involve children in mealtime preparation.

While the home environment is often the primary eating environment for children, especially during infancy (19), children are also exposed to eating environments outside the home. Children who attend day-care settings are frequently exposed to new foods and to the eating behaviour of peers and other adults. Experimental studies involving live trained peers (46) or orchestrated video recordings of peers (47–49) have shown that peer modelling can be conducted to increase vegetable consumption in preschool and school settings. The potential for teachers and early years educators to function as effective role models to support healthy eating is more mixed (50–52) and may depend on the adult engaging in highly enthusiastic modelling (53). Nevertheless, it is clearly important not to ignore out-of-home environments as potential influencers of children’s vegetable consumption and as settings for healthy eating interventions (54).

Children’s eating behaviours and food preferences are not only a product of their environment but are also subject to individual differences between children themselves. During toddlerhood, as children start to show autonomy over their food choices, individual differences become apparent in children’s willingness to try new foods and in their selectivity over the foods they will eat. Between18 and 58 months of age, children differ in the extent to which they might be classed as ‘fussy’ eaters (55), with fussiness defined as eating selectively, being picky about what is eaten, and refusing to eat both familiar and unfamiliar foods. Food fussiness is thought to particularly impact vegetable acceptance because the bitter flavours and softer textures of vegetables render these less palatable than other foods (56). Food fussiness can occur in conjunction with food neophobia, the more common tendency to avoid new foods; both peak between 2 and 6 years of age (56–59). There is some evidence that fussy eating is associated with gender. While some studies have found no gender differences in eating behaviours in early childhood (60, 61), others report more picky and selective eating among boys (62–64). Boys have also been reported to consume fewer vegetables than girls in early childhood (23), a trend that is seen to strengthen as children get older (65).

Child temperament is also related to eating behaviour (40, 55). Children with shy or more emotional temperaments display more food avoidant behaviours (60, 66, 67) and are more neophobic (68), and children who are considered anxious or dependent are less likely to consume a healthy diet (69). Negative temperament traits have also been found to be associated with higher BMI in infants, preschoolers, and older children (40, 70). More challenging infant temperaments have been linked to negative mealtime experiences, and negative traits that track into adulthood have been linked to maladaptive eating behaviours (71), suggesting a pathway from early temperament to negative health outcomes (72).

It is important to note that parent and child factors are not independent of one another. Child temperament is, to some extent, a consequence of parenting style, while parents’ feeding practices are, in part, a response to the child’s temperament and willingness to accept the foods offered (66). Children who are considered to be difficult or to have a negative temperament are more likely to be soothed and calmed with food by their parents (73, 74), while more indulgent feeding styles are used by parents of children with higher levels of negative affect (75). Similarly, parents of children who are considered to be fussy or picky eaters often report mealtimes to be challenging (72, 76) and that they avoid offering foods likely to be rejected by their child as a result (76). Evidence shows that the familiarity brought about by repeated exposure to a vegetable is a powerful mechanism for supporting willingness to try vegetables and acceptance of these into the child’s diet (77–79). Parents’ avoidance of rejected foods is therefore likely to exacerbate the problem both by limiting the opportunities for a food to become familiar and by reinforcing the child’s behaviour in rejecting it. In other cases, food fussiness can cause parents to adopt more authoritarian feeding practices (45), which may be maladaptive in achieving the goals they are aiming for at mealtimes (80). Thus, children’s eating behaviour in general, and their vegetable intake in particular, may depend on a complex interplay between factors that are individual to the child and to the parent, including the parent’s motivations around family mealtimes.

While previous studies have established differences in the quality of children’s diets (11) and in rates of childhood overweight and obesity (10) in different parts of Europe, there has been no investigation to date of whether the environmental, parental, and individual child factors that predict vegetable intake differ in different populations of preschool children. Furthermore, previous cross-country comparisons have focussed on the early infancy (41) and adolescent periods (81) when, as discussed above, the preschool years are a critical period in the development and formation of food preferences and a prime target for interventions to support healthy eating behaviour.

This study draws together several previously distinct lines of enquiry from cognitive and health psychology, epidemiology, and nutrition science to establish the environmental, parent, and child factors associated with children’s vegetable intake and the extent to which these vary across populations. Data were collected in three countries (Italy, Poland, and the UK) in distinct regions of Europe, selected to represent a cross-section of the geographical and cultural landscapes of children’s dietary quality, as discussed above (10–12). Parents of preschool children aged between 18 and 48 months were invited to complete a range of questionnaire measures about the family’s demographics, the parent’s feeding practices, the family’s mealtime goals, the child’s temperament, and the child’s food fussiness, allowing us to explore how these factors combine to predict levels of vegetable consumption. Our research questions were as follows:

(1) Do preschool children in Italy, Poland, and the UK differ in the extent to which they meet WHO guidelines on recommended levels of vegetable intake?
(2) Do the environmental, parental, and individual child factors previously implicated in children’s eating behaviour differ between countries and in their association with vegetable intake in each country?
(3) Are the environmental, parent, and child factors that combine to predict preschooler’s vegetable intake stable across populations?

## Materials and methods

### Design

The participating parents of preschoolers completed a set of online questionnaires that asked about the child’s and family’s demographic characteristics including child’s age, gender, ethnicity, attendance at day care, parents’ education (as a proxy for socioeconomic status), relationship to child, number of children in the home, parents’ feeding style and mealtime goals, and the child’s eating behaviour, temperament, and vegetable intake. After completing the questionnaires, a subset of the participants in each country took part in an intervention to increase children’s vegetable consumption; the results of which are reported elsewhere (82, 83). The study was conducted across three sites in Italy, Poland, and the UK. Data collection methods were very similar across sites but were adapted where necessary to meet local research ethics requirements or for practical reasons specific to a site. The design of the intervention study and sample size calculation were pre-registered^1^.

The study received ethical approval from each country’s designated ethics committee (UK: University of Reading Research Ethics Committee, approval no. 2019-018-CHP, date 19 March 2019; Italy: Ethics Committee of the University of Turin, approval no. 176852, date 2 February 2019; Poland: Research Ethics Committee at the Faculty of Psychology, University of Warsaw, date 8 April 2020).

### Participants

The participants were parents of preschool children aged between 18 and 48 months. They were recruited by researchers in Italy, Poland, and the UK and their partner organisations between September 2019 and December 2020. Families were recruited *via* online channels (social media, web pages, and press sites), *via* face-to-face contact at sites where families congregate (e.g., kindergartens, sports centres, and bus stops), and *via* promotional activity through existing partner networks including *Szkołanawidelcu* (School on a Fork) in Poland, and the British Nutrition Foundation and the University of Reading’s Child Development Group database in the UK.

Informed written consent was obtained from all the participants. In Italy, to adhere to local research ethics requirements, both parents provided written consent in hard copy. In Poland and the UK, consent was provided electronically prior to completing the questionnaires. Correspondence with families was by email. Communication with the participants was, in all cases, in the language of the recruiting country (Italian, Polish, or English).

A total of 410 parents consented to participate and completed the questionnaires (Italy: *N* = 61, Poland: *N* = 124, and the UK: *N* = 225). G*Power analyses suggested a target sample of 450 participants (150 per country), which, allowing for attrition over the course of the study, should provide 128 cases per group. Differences in final sample sizes resulted from variations in the duration of active recruitment and differences in recruitment success rates. Several families with children outside the target age range were keen to participate. To maximise the sample size, eligibility was extended to include parents of children aged between 16 and 58 months; data from two further families with children younger than 16 months were excluded. The mean age of children in the final sample (*N* = 408) was 32.2 months (*SD* = 9.5 months) (Italy: mean = 35 months, *SD* = 10.6; Poland, mean = 32.5 months, *SD* = 9.7; UK: mean = 31.3 months, *SD* = 8.9).

### Materials

Data collection was conducted online using survey platforms available to the local research team (in Italy: Google Forms; in Poland: Qualtrics; in UK: a purpose-built study website). The survey questions comprised both validated scales used in previous eating behaviour research and questionnaires created specifically for the purposes of the study. Original measures were available only in English; members of the Italian and Polish research teams arranged for manual translation of all measures into their respective languages.

#### Demographic measures

The participants were asked the following about their child and family: child’s date of birth (from which child age was calculated), child’s gender (male/female), child’s ethnicity (in UK, the categories were those used in the 2011 census (84); in Poland, the categories were White/African/Asian/I do not know/I prefer not to answer/Other; in Italy, it was not deemed appropriate to ask about ethnicity), attendance at day care (yes/no), number of children living at home, whether the child was the first born, country of residence, relationship of the responding parent to the child (mother/father/other), and educational level of both parents (categories were no formal education/school education equivalent to GCSE level in UK/vocational qualification/high school education equivalent to A-level/bachelor’s degree/higher degree). As the sample was predominantly highly educated, the first three categories were combined for analysis purposes.

#### Child vegetable intake

##### Vegetable food frequency questionnaire

The parents were asked to indicate whether their child had eaten each of the vegetables on the vegetable food frequency questionnaire (VegFFQ) during the preceding 2 weeks. This measure was based on an instrument used in previous research (85) but was adapted to include vegetables common to the country in which it was being used, in consultation with professional nutritionists. The VegFFQ, therefore, differed across versions both in the number of vegetables (Italy: *N* = 24, Poland: *N* = 27, and UK: *N* = 24) and the specific vegetables listed (refer to Appendix). The parents were asked to report how frequently during the previous 2 weeks their child had eaten a child-sized portion of each of the vegetables on a five-point scale (categories were: never/once/a few times/many times/every day). A child-sized portion was defined for the parents as the amount that fits in a child’s hand, in line with UK guidance (14).

To compute the average number of portions of vegetables children ate per day during the period in question, the ratings were recoded as follows: ‘never’ = 0, ‘once’ = 1, ‘a few times’ = 3, ‘many times’ = 6, and ‘every day’ = 14, converting fortnightly ratings of frequency to the values these implied. To adjust for the different numbers of vegetables on each country’s list and the inclusion of potatoes on the Italian and Polish lists [potatoes do not count toward the recommended 5 portions of vegetables per day in the UK guidance (14)], we selected the 23 most frequently eaten vegetables in each country after excluding potatoes (refer to Table 1). Scores for the selected 23 vegetables were summed for each child and divided by 14 to compute the mean portions consumed per day, which was used as the measure of vegetable intake.

**TABLE 1:** Mean child-sized portions of vegetables eaten per day by children in the UK, Italy, and Poland.

	UK	Italy	Poland
*Mean (SD)*	*N* = 225	*N* = 61	*N* = 124
Veg intake (child portions per day)	2.48 (1.66)	1.72 (1.06)	3.14 (2.03)
Variety of intake (number of different veg eaten)	9.96 (4.53)	8.62 (3.68)	12.52 (5.44)
Top 23 vegetables eaten by children in each country, listed from most to least frequently consumed	Cucumber, Carrots, Tomato, Peas, Sweetcorn, Broccoli, Peppers, Green Beans, Sweet Potato, Courgette, Mushroom, Cauliflower, Spinach, Lettuce, Butternut Squash, Leeks, Parsnip, Cabbage, Broad Bean, Beetroot, Aubergine, Asparagus, Brussels Sprouts.	Carrots, Courgette, Tomato, Peas, Butternut Squash, Fennel, CherryTomatoes, Green Beans, Lettuce, Spinach, Broccoli, Cauliflower, Chard, Aubergine, Leeks, Cucumber, Peppers, Cabbage, Artichoke, Brussels Sprouts, Broad Beans, Asparagus, Beetroot.	Tomato, Cucumber, Carrots, Onion, Peppers, Courgette, Parsley root, Broccoli, Sweetcorn, Green Beans, Cauliflower, Peas, Beetroot, Leeks, Spinach, Lettuce, Radish, Broad Bean, Cabbage, Mushroom, Pumpkin, Brussels Sprouts, Asparagus.

Variety of vegetable intake was assessed in terms of the number of different vegetables children were reported to have eaten during the 2-week period of interest, out of the same set of 23 vegetables included in computations of vegetable intake.

#### Parent measures

##### Family Mealtime Goals questionnaire

The Family Mealtime Goals [FMG (31)] questionnaire asks parents about the goals they have in mind when planning family meals and has been shown to have good psychometric properties. Items are scored on a five-point scale from ‘strongly agree’ (1) to ‘strongly disagree’ (5) and reverse-scored where appropriate. Questions related to three of the instrument’s eight dimensions were used in this study to ensure that the overall length of the survey was manageable for parents; the dimensions selected were those most likely to be relevant to children’s vegetable consumption and comprised Shared Family Food, Stress/Conflict Avoidance, and Family Involvement in Mealtimes. Examples of items are: “I don’t want to prepare different foods for different family members” and “I want to avoid arguments at mealtimes.” Scores were calculated by summing scores for questions related to each component.

##### Comprehensive Feeding Practices Questionnaire

The Comprehensive Feeding Practices Questionnaire [CFPQ (86)] comprises 49 items assessing parent feeding style and has been shown to have good psychometric properties. The questionnaire comprises 12 subscales: Monitoring, Emotional Regulation, Food as Reward, Child Control, Modelling, Restriction for Weight, Restriction for Health, Teaching Nutrition, Encourage Balance, Pressure to Eat, Healthy Environment, and Involvement. The items are rated on a five-point scale from ‘never’ (1) to ‘always’ (5) or from ‘disagree’ (1) to ‘agree’ (5) and reversed-scored as required. Examples of questions are: “How much do you keep track of the sweets (candy, ice cream, cake, pies, and pastries) that your child eats?” and “I allow my child to help prepare family meals.” The CFPQ was provided to parents in the UK and Italy as an additional measure and was completed by 230 participants in these countries. This measure was not administered in Poland as it was considered that the additional time required to complete the instrument would impact negatively on participant engagement.

#### Child measures

##### Child Eating Behaviour Questionnaire: Food Fussiness subscale

Individual differences in children’s food fussiness were assessed using the Food Fussiness subscale of the Child Eating Behaviour Questionnaire [CEBQ-FF (87)], which has been shown to have good psychometric properties. The subscale comprises six items rated on a five-point scale from ‘never’ (1) to ‘always’ (5), which are reverse-scored as required. Example questions are “My child enjoys a wide variety of foods” and “My child decides that s/he doesn’t like food, even without tasting it.” A mean score was calculated for each participant, with higher values indicating greater fussiness.

##### Emotionality Activity Sociability Scale – Temperament subscale

Child temperament was measured using the Temperament subscale of the Emotionality Activity Sociability Scale [EAS-T; (88)], which has been shown to have good psychometric properties. This questionnaire consists of 18 items rated on a 5-point scale from ‘not typical of my child’ (1) to ‘very typical of my child’ (5), which are reverse-scored as required. The questionnaire comprises four dimensions: Activity, Emotionality, Shyness, and Sociability. Examples of items are “My child cries easily” and “My child likes to be with people.” This questionnaire was provided to parents in the UK and Italy as an additional measure and was completed by 230 parents. This measure was not administered in Poland for the same reasons outlined above.

### Procedure

After parents had given their consent to participate, they were sent a link to the online questionnaire, which presented measures in this fixed order: demographic questions, food fussiness (CEBQ-FF), vegetable consumption (VegFFQ), and parents’ mealtime goals (FMGs). Additional measures to assess child temperament (EAR-T) and parent feeding practices (CFPQ) were completed by all the participants in Italy and included as optional additional questionnaires for parents in the UK. Upon completion, the parents were thanked for their time and were invited to participate in an intervention to support vegetable intake, the results of which reported elsewhere (82, 83).

### Approach to data analysis

The data were analysed using statistical software SPSS version 26 (89). For standardised questionnaires, summary measures (mean or total scores) were calculated as described by the questionnaire’s authors. For measures developed for the purposes of this study, scoring was as described in the Materials section above. As is common for questionnaire data, measures were frequently non-normally distributed. Parametric and non-parametric tests were therefore conducted in parallel to check for discrepancies in outcomes; in almost all cases, the results of these matched, and we report the results of parametric tests in the text given their easier interpretation. When discrepancies in findings were seen, we additionally report the results of the non-parametric comparisons in a footnote. Between-country comparisons of the demographic variables and parent and child measures involved analyses of variance with Bonferroni corrected *post hoc* tests, *t*-tests (and their non-parametric equivalents), and chi-squared analyses depending on the nature of the data and the number of countries contributing data. Pearson’s correlations, analyses of variance, or *t*-tests (and their non-parametric equivalents) were conducted to explore the relationships between each of the demographic, parent, and child measures with children’s vegetable intake, again followed up by *post hoc* tests as required. Multiple linear regression was conducted to establish the combined predictive value of factors found to be associated with vegetable intake in each country separately and in combined models that included predictor-country interactions. To assess and compare model fit, Fisher’s *Z* tests were conducted.

## Results

We first describe children’s reported vegetable intake in each country, the dependent variable of interest in this study, and explore differences in levels of consumption across the three samples. We then report the environmental, parent, and child factor measures collected, explore differences in these between the samples, and examine whether these measures are related to and help to predict individual differences in children’s intake of vegetables in each country.

### Comparisons of children’s vegetable intake across countries

Table 1 presents the reported vegetable consumption of children in each country in terms of mean child-sized portions consumed per day, the number of different vegetables eaten during the period of assessment, and the specific vegetables eaten in each country in order of frequency. Children in all three groups ate significantly less than five portions of vegetables per day [UK: *t*(224) = –22.72, *p* < 0.001; Italy: *t*(60) = –24.1, *p* < 0.001; Poland*: t*(121) = –10.12, *p* < 0.001]. However, vegetable intake differed significantly between groups, *F*(2,405) = 14.52, *p* < 0.001, η*^2^* = 0.07. The Polish children consumed more portions per day than the children in the UK [*t*(345) = –3.23, *p* = 0.001] and Italy [*t*(180.6) = –6.19, *p* < 0.001], while the children in UK ate more vegetables than those in Italy [*t*(148.32) = 4.35, *p* < 0.001]. The children in the UK and Italy both ate significantly less than three portions per day [UK: *t*(224) = –4.66, *p* < 0.001; Italy*: t*(60) = –9.4, *p* < 0.001], the level recently found to be optimal for good health (90).

We also examined the variety of vegetables consumed in each country in terms of the number of different vegetables children were reported to have eaten during the 2-week period of interest. Variety of intake also differed significantly between groups, *F*(2,405) = 17.42, *p* < 0.001, η*^2^* = 0.08, with a pattern very similar to that seen for vegetable intake. The Polish children consumed a greater variety of vegetables than the children in the UK [*t*(212.78) = –4.41, *p* < 0.001] and Italy [*t*(165.07) = –5.71, *p* < 0.001], and the children in the UK ate a wider variety of vegetables than the children in Italy [*t*(284) = 2.13, *p* = 0.03]. Variety of intake was highly correlated with quantity of vegetable intake (portions per day), both overall [*r*(408) = 0.82, *p* < 0.001] and in each country separately [UK: *r*(225) = 0.8, *p* < 0.001; Italy: *r*(61) = 0.71, *p* < 0.001; Poland: *r*(122) = 0.83, *p* < 0.001].

Similarities were observed between the groups in terms of the specific vegetables eaten. Carrots and tomatoes were among the most frequently eaten vegetables, and asparagus and Brussels sprouts were among the least frequently eaten vegetables in each country. There were also noteworthy differences. For example, cucumber was very commonly eaten in Poland and the UK but was rarely eaten by children in Italy.

### The environmental, parent, and child characteristics of each group and their relationship with vegetable intake

#### Environmental/sociodemographic characteristics

The sociodemographic data for the participants from each country are shown in Table 2. There were several between-country differences in sociodemographic characteristics including child age [*F*(2,405) = 3.88, *p* = 0.02, η^2^ = 0.02]. *Post hoc* comparisons showed that the children of the Italian participants were slightly older than those of the UK participants. The Italian sample had more male children than the other groups, although the distribution did not differ significantly between countries [χ*^2^*(2) = 5.12, *p* = 0.08]. There was a between-country difference in the proportion of children attending day care (χ*^2^*(2) = 39.21, *p* < 0.001]; fewer children attended day care in the Polish sample than in the samples from Italy and the UK. There were no significant differences between the groups in the number of children in the family or in whether participating children were the first born. Educational levels differed between countries for both parents [parent 1: Fisher’s exact tes*t p* < 0.001; parent 2: χ*^2^*(6) = 69.31, *p* < 0.001]; the Polish parents were the most highly educated, followed by the Italian parents, followed by the British parents. Ethnicity was measured differently at each site and was not collected in Italy, preventing direct comparison. However, the large majority of parents identified their children as ‘White’ in the UK (95%) and Poland (93%). Finally, while the majority of participants in each sample resided in the country in which they were recruited to the study, in each case, a small number lived elsewhere in the world. For ease of reporting, we refer to our samples as Italian, Polish, and British, respectively.

**TABLE 2:** Participant demographic characteristics.

	UK	Italy	Poland
*N*	225	61	122
Child age (months), *M* (*SD*)	31.3 (8.9)	35.0 (10.6)	32.5 (9.7)
Child gender, % male	49.3	65.6	51.6
*N* children in home, %			
1	43.6	44.3	46.7
2	47.6	41.0	42.6
3+	6.7	14.8	10.6
Child is first born, % yes	60.9	54.1	68.9
Attends day care, % yes	80.0	88.5	52.5
Relationship to child, %			
Mother	96.0	93.4	98.4
Father	3.6	6.6	0.8
Other	0.4	0	0.8
Education of Parent 1, %			
No formal education/GCSE-	5.3	1.6	3.3
level/vocational qualifications			
High-school education	10.7	23.0	0.0
Bachelor’s education	39.4	18.0	11.5
Higher degree education	44.4	55.7	85.2
Education of Parent 2, %			
No formal education/GCSE-	17.3	6.6	26.2
level/vocational qualifications			
High-school education	16.9	31.1	0.0
Bachelor’s education	34.7	9.8	18.9
Higher degree education	29.3	45.9	54.9
Ethnicity of child, %			
White	–	–	97.5
White British	81.8	–	–
White and Asian	3.1	–	–
White and Black African	1.3	–	–
Bangladeshi	0.4	–	–
Pakistani	0.4^3^	–	–
Indian	0.4	–	–
Irish	0.9	–	–
Chinese	0.4	–	–
Arab	0.4	–	–
White –other^a^	6.7	–	–
Mixed –other^b^	2.2	–	–
Prefer not to say	1.8	–	0.8
Other^c^	–	–	1.6
Country of Residence, %			
Italy	–	96.7	–
UK	95.5	1.6	0.8
Poland	0.4	–	93.4
Australia	1.8	–	–
South Africa	–	1.6	–
Holland	–	–	1.6
Germany	0.9	–	1.6
Ireland	0.9	–	–
Norway	–	–	0.8
Netherlands	–	–	0.8
Jordan	–	–	0.8
Mauritius	0.4	–	–

^a^White-Other included Hungarian/Irish/English, European, Australian, French, Russian/Scottish, Slavic, Russian, Polish/English, Latino of European descent, Italian/Turkish Cypriot, German, New Zealand, and European.

^b^Mixed-Other included White/Fijian, English/Colombian, Indian/Chinese, White/African Arab, Portuguese Black, Caribbean, and Asian.

^c^“Other” included White and Asian.

Next, we examined whether any sociodemographic characteristics (child’s gender, age, day care attendance, birth order, parents’ education level, and number of children in the home) were associated with children’s vegetable intake, either for the group overall or in any individual country. In the group as a whole, children who attended day care were reported to consume fewer portions of vegetables (*M* = 2.42, *SD* = 1.68) than children solely cared for at home (*M* = 2.97, *SD* = 1.93) [*t*(406) = 2.84, *p* = 0.005, Cohen’s *d* = 0.32]. However, the effect of day care attendance was not significant for any individual sample. The education level of the responding parents was significantly associated with children’s vegetable intake, *F*(3,402) = 3.5, *p* = 0.016, η*^2^* = 0.03*; post hoc* tests showed that the children of parents with higher degrees had higher intake of vegetables (*M* = 2.79, *SD* = 1.86) than the children of parents holding bachelor’s degrees (*M* = 2.15, *SD* = 1.62). The same general pattern was seen in the UK sample, *F*(3,220) = 3.09, *p* = 0.028, η*^2^* = 0.04, although *post hoc* tests found no significant differences in the vegetable intake of children whose parents fell in different educational categories. There were no other significant associations between the demographic measures collected and children’s vegetable intake, including no relationships with age (*p* > 0.05 for all the analyses).

#### Parent mealtime goals and feeding style

Table 3 presents the mean scores for each component of the Family Mealtime Goals (FMG) questionnaire. Significant between-country differences were found for endorsement of the ‘shared family food’ goal [*F*(2,405) = 9.88, *p* < 0.001, η*^2^* = 0.05]. The parents in the UK and Poland endorsed this goal more strongly than the parents in Italy [Italy vs. UK*: t*(284) = 3.43, *p* = 0.001; Italy vs. Poland*: t*(181) = –4.51, *p* < 0.001. There was no significant difference in the goal’s endorsement in the UK and Poland: *t*(345) = –1.67, *p* = 0.1]. The same pattern was seen for the goals of ‘stress and conflict avoidance’ [*F*(2,405) = 7.83, *p* < 0.001,η^2^ = 0.04] [Italy vs. UK: *t*(284) = 3.74, *p* < 0.001; Italy vs. Poland: *t*(181) = –3.53, *p* = 0.001*;* UK vs. Poland: *t*(345) = –0.05, *p* = 0.96] and ‘family involvement in mealtimes’ [*F*(2,405) = 8.62, *p* < 0.001, η^2^ = 0.04] [Italy vs. UK: *t*(284) = 3.39, *p* = 0.001*;* Italy vs. Poland: *t*(181) = –4.25, *p* < 0.001*;* UK vs. Poland: *t*(345) = –1.25, *p* = 0.21]. In each case, the UK and Polish parents endorsed the goals more strongly than the Italian parents.

**TABLE 3:** Family mealtime goals questionnaire component scores for parents in the UK, Italy, and Poland.

	Whole sample	UK	Italy	Poland
*N*	408	225	61	122
FMG component	*M (SD)*	*M (SD)*	*M (SD)*	*M (SD)*
Shared family food	4.44 (0.66)	4.45 (0.66)	4.12 (0.70)	4.57 (0.61)
Stress/Conflict avoidance	4.44 (0.65)	4.50 (0.64)	4.15 (0.68)	4.50 (0.62)
Family involvement	4.04 (0.71)	4.06 (0.72)	3.72 (0.69)	4.16 (0.66)

Table 4 provides the scores for the British and Italian participants for each component of the Comprehensive Feeding Practices Questionnaire (CFPQ) (this measure was not collected in Poland). The two groups differed on their endorsement of the feeding behaviours of ‘emotion regulation’ [*t*(228) = 2.94, *p* = 0.004] and ‘modelling’ [*t*(224) = 3.33, *p* = 0.001]. In both cases, the British parents reported engaging in these behaviours to a greater extent than the Italian parents.

**TABLE 4:** Comprehensive feeding practices questionnaire component scores for parents in the UK and Italy.

	UK	Italy
*N*	169	61
CFPQ component	*M (SD)*	*M (SD)*
Child control	2.54 (0.61)	2.69 (0.72)
Emotion regulation	2.02 (0.71)	1.70 (0.73)
Encourage balance and variety	4.28 (0.58)	4.36 (0.57)
Environment	3.91 (0.72)	3.98 (0.73)
Food as reward	2.04 (0.93)	2.21 (1.16)
Involvement	3.07 (1.05)	3.15 (0.97)
Modelling	4.24 (0.68)	3.89 (0.77)
Monitoring	4.21 (0.76)	4.35 (0.65)
Pressure	2.71 (0.94)	2.86 (0.91)
Restrictions for health	3.10 (1.04)	2.98 (1.05)
Restrictions for weight	1.82 (0.59)	1.97 (0.59)
Teaching about nutrition	3.65 (0.97)	3.61 (0.89)

To explore whether these parenting factors were related to children’s vegetable intake, correlational analyses were conducted between the component measures of the two parent measures and children’s mean vegetable intake per day. When all the participants were included in analyses (refer to Table 5), endorsement of the Family Mealtime Goals (FMG) component of ‘family involvement’ [*r*(408) = 0.18, *p* < 0.001] was positively associated with vegetable intake. Vegetable intake was also related to two components from the CFPQ: endorsement of the ‘healthy environment’ dimension was positively related to vegetable intake [*r*(229) = 0.15, *p* = 0.03], while endorsement of the use of ‘food as a reward’ was negatively related to vegetable intake [*r*(229) = –0.2, *p* = 0.002].

**TABLE 5:** Correlations between vegetable consumption and parent and child measures, all children included (*N* = 408)^**†**^.

Variables	1	2	3	4	5	6	7	8	9	10	11	12	13	14	15	16	17	18	19	20	21
(1) Portions of veg per day	–																				
(2) Child’s age	–0.06	–																			
(3) Food fussiness	–0.28**	0.19**	–																		
(4) EAS-T activity	0.01	–0.09	–0.05	–																	
(5) EAS-T emotionality	0.05	0.05	0.20**	–0.10	–																
(6) EAS-T shyness	–0.10	0.01	0.19**	–0.33**	0.30**	–															
(7) EAS-T sociability	–0.03	0.08	–0.11	0.21**	0.12	–0.27**	–														
(8) CFPQ child control	–0.11	0.05	0.17*	–0.04	0.14*	0.14*	–0.03	–													
(9) CFPQ emotion regulation	0.05	–0.05	0.04	–0.15*	0.12	0.06	–0.01	0.19**	–												
(10) CFPQ encourage balance and variety	0.08	0.09	–0.06	0.11	0.02	0.02	–0.05	–0.01	–0.08	–											
(11) CFPQ environment	0.15*	–0.01	0.01	–0.01	–0.02	–0.03	0.05	–0.12	–0.14*	0.25**	–										
(12) CFPQ Food as reward	–0.20**	0.27**	0.16*	0.04	0.06	–0.06	–0.04	0.07	0.33**	0.11	–0.23**	–									
(13) CFPQ involvement	0.08	0.35**	0.03	0.05	–0.06	0.00	–0.02	0.05	–0.01	0.31**	0.21**	0.07	–								
(14) CFPQ modelling	0.05	0.02	0.12	0.00	–0.08	0.04	–0.10	–0.09	0.07	0.41**	0.34**	0.01	0.27**	–							
(15) CFPQ monitoring	0.02	–0.05	–0.09	0.05	0.03	–0.00	0.03	–0.20**	–0.13	0.22**	0.29**	–0.19**	0.11	0.25**	–						
(16) CFPQ pressure	0.03	0.07	0.04	–0.02	0.06	–0.05	–0.05	–0.11	0.09	0.20**	–0.15*	0.37**	0.03	0.06	–0.06	–					
(17) CFPQ restriction for health	–0.02	0.17*	0.09	0.03	0.12	–0.04	0.03	–0.04	0.21**	0.18**	–0.16*	0.36**	0.11	0.13*	–0.07	0.37**	–				
(18) CFPQ restriction for weight control	–0.04	0.03	–0.08	0.11	0.08	–0.12	0.06	–0.11	0.06	0.13	–0.06	0.24**	0.06	0.03	0.11	0.29**	0.41**	–			
(19) CFPQ teaching about nutrition	0.07	0.34**	0.07	0.03	0.02	–0.03	–0.04	–0.06	–0.00	0.38**	0.14*	0.10	0.52**	0.31**	0.25**	0.06	0.21**	0.17**	–		
(20) FMGQ shared family food	0.07	–0.02	–0.02	–0.06	–0.00	0.02	–0.06	–0.18**	–0.04	–0.04	–0.10	0.02	–0.15*	0.06	–0.06	0.03	–0.01	–0.02	–0.01	–	
(21) FMGQ stress/conflict avoidance	0.05	–0.02	0.06	–0.05	0.07	0.11	–0.02	–0.03	0.07	–0.07	–0.07	–0.03	–0.10	0.01	–0.04	0.07	0.06	0.04	0.02	0.42**	–
(22) FMGQ family involvement at mealtimes	0.18**	0.16**	0.01	0.01	0.03	0.03	–0.00	–0.02	0.05	0.05	0.12	0.05	0.40**	0.24**	0.06	–0.02	0.13	–0.01	0.29**	0.31**	0.33**

*p < 0.05, **p < 0.01.

EAS-T, Emotionality Activity Sociability-Temperament subscale (88); CFPQ, Comprehensive Feeding Practices Questionnaire (86); FMGQ, Family Mealtime Goals questionnaire (31).

^†^CFPQ and EAS-T were completed by a subset of the population (*N* = 230).

To explore whether the parent factors were associated with vegetable consumption in all three countries, analyses were conducted for each country separately (refer to Tables 6–8). For the participants in the UK, the CFPQ ‘environment’ [*r*(168) = 0.17, *p* = 0.028] and ‘food as a reward’ components [*r*(168) = --0.18, *p* = 0.02] and the FMG dimension ‘family involvement’ [*r*(225) = 0.17, *p* = 0.01] were associated with children’s vegetable intake^2^. In the Italian sample, in addition to a significant association with ‘food as a reward’ [*r*(61) = –0.29, *p* = 0.02], vegetable intake was negatively associated with the CFPQ dimensions of ‘emotion regulation’ [*r(*61) = –0.29, *p* = 0.02] and ‘food restriction on health’ [*r*(61) = –0.27, *p* = 0.03]^2^. In the Polish sample where only the Family Mealtime Goals questionnaire was collected, no parenting component was significantly associated with vegetable intake.

**TABLE 6:** Correlations between vegetable consumption and parent and child measures, UK sample (*N* = 225)^**†**^.

Variables	*n*	1	2	3	4	5	6	7	8	9	10	11	12	13	14	15	16	17	18	19	20	21
(1) Portions of veg per day	225	–																				
(2) Child’s age	225	–0.01	–																			
(3) Food fussiness	225	–0.29**	0.08	–																		
(4) EAS activity subscale	169	0.03	–0.03	–0.01	–																	
(5) EAS emotionality subscale	169	0.15	–0.05	0.16*	–0.09	–																
(6) EAS shyness subscale	169	–0.05	–0.02	0.16*	–0.36**	0.29**	–															
(7) EAS sociability subscale	169	0.05	–0.02	–0.04	0.22**	0.16*	–2.4**	–														
(8) CFPQ child control	169	–0.07	0.04	0.19*	–0.01	0.08	0.10	–0.01	–													
(9) CFPQ emotion regulation	169	0.07	–0.08	–0.07	–0.11	0.08	0.04	0.12	0.16*	–												
(10) CFPQ encourage balance and variety	169	0.12	0.12	–0.05	0.10	0.07	0.05	–0.05	0.03	–0.04	–											
(11) CFPQ environment	168	0.17*	0.04	0.03	–0.03	0.00	0.01	0.01	–0.05	–0.08	0.25**	–										
(12) CFPQ food as reward	168	–0.18*	0.29**	0.09	0.06	–0.04	–0.11	0.02	0.03	0.25**	0.13	–0.20**	–									
(13) CFPQ involvement	168	0.10	0.42**	–0.01	–0.00	–0.06	0.01	–0.05	0.13	0.02	0.32**	0.19*	0.08	–								
(14) CFPQ modelling	165	0.06	0.10	0.07	–0.06	–0.04	0.05	–0.02	0.03	0.03	0.38**	0.45**	–0.01	0.30**	–							
(15) CFPQ monitoring	169	0.06	–0.05	–0.12	0.02	0.07	0.02	–0.02	–0.18*	–0.10	0.18*	0.24**	–0.17*	0.05	0.20**	–						
(16) CFPQ pressure	168	0.10	0.08	0.05	–0.06	0.10	0.03	–0.06	–0.18*	0.05	0.17*	–0.09	0.34**	0.04	–0.01	–0.03	–					
(17) CFPQ restriction for health	168	0.02	0.16*	–0.02	0.06	0.11	–0.04	0.14	–0.02	0.16*	0.23*	–0.14	0.36**	0.04	0.09	–0.10	0.38**	–				
(18) CFPQ restriction for weight control	168	0.02	0.05	–0.08	0.08	0.11	–0.08	0.07	–0.11	0.05	0.14	–0.05	0.21**	–0.01	0.01	0.15	0.29**	0.41**	–			
(19) CFPQ teaching about nutrition	168	0.07	0.42**	–0.01	0.05	0.03	–0.01	0.01	–0.01	–0.00	0.37**	0.18*	0.11	0.54**	0.33**	0.25**	0.03	0.16*	0.15	–		
(20) FMGQ shared family food	225	0.02	0.04	–0.01	–0.03	–0.02	–0.01	0.08	–0.18*	–0.11	0.02	–0.07	0.03	–0.09	0.08	0.01	0.04	0.01	–0.03	0.02	–	
(21) FMGQ stress/conflict avoidance	225	0.03	0.00	–0.02	–0.03	0.10	0.06	0.13	–0.13	0.03	–0.03	–0.12	–0.00	–0.15*	–0.07	–0.02	0.12	0.10	0.08	0.04	0.49**	–
(22) FMGQ family involvement at mealtimes	225	0.17**	0.24**	–0.08	0.01	0.03	–0.00	0.12	0.08	–0.03	0.08	0.12	0.01	0.38**	0.19*	0.03	0.00	0.06	–0.07	0.32**	0.39**	0.30**

*p < 0.05, **p < 0.01.

EAS-T, Emotionality Activity Sociability-Temperament subscale (88); CFPQ, Comprehensive Feeding Practices Questionnaire (86); FMGQ, Family Mealtime Goals questionnaire (31).

^†^CFPQ and EAS-T were completed by a subset of the UK sample (*N* = 168).

**TABLE 7:** Correlations between vegetable consumption and parent and child measures, Italian sample (*N* = 61).

Variables	1	2	3	4	5	6	7	8	9	10	11	12	13	14	15	16	17	18	19	20	21
(1) Portions of veg per day	–																				
(2) Child’s age	0.13	–																			
(3) Food fussiness	–0.27**	0.40**	–																		
(4) EAS activity	–0.09	–0.29*	–0.15	–																	
(5) EAS emotionality	–0.26*	0.26*	0.42**	–0.18	–																
(6) EAS shyness	–0.36**	0.10	0.25*	–0.23	0.37**	–															
(7) EAS sociability	0.10	0.10	–0.11	0.24	–0.16	–0.41**	–														
(8) CFPQ child control	–0.19	0.20	0.17	–0.14	0.26*	0.22	–0.18	–													
(9) CFPQ emotion regulation	–0.29*	0.11	0.17	–0.28*	0.41**	0.11	–0.07	0.32	–												
(10) CFPQ encourage balance and variety	–0.02	0.01	–0.08	0.17	–0.19	–0.06	–0.12	–0.12	–0.14	–											
(11) CFPQ environment	0.15	–0.15	–0.02	0.07	–0.15	–0.12	0.08	–0.30*	–0.26*	0.25	–										
(12) CFPQ food as reward	–0.29*	0.20	0.30*	–0.02	0.30*	0.05	–0.24	0.13	0.58*	0.07	–0.30*	–									
(13) CFPQ involvement	0.00	0.17	0.12	0.24	–0.07	–0.02	–0.01	–0.16	–0.05	0.26*	0.25	0.04	–								
(14) CFPQ modelling	–0.22	–0.03	0.12	0.21	–0.08	0.01	–0.04	–0.26*	0.03	0.59**	0.15	0.11	0.23	–							
(15) CFPQ monitoring	–0.06	–0.10	0.00	0.14	–0.18	–0.07	0.05	–0.30*	–0.17	0.37**	0.44**	–0.28*	0.31*	0.47**	–						
(16) CFPQ pressure	–0.15	0.01	0.06	0.14	–0.11	–0.28*	–0.13	0.01	0.27*	0.26*	0.34**	0.45**	–0.02	0.27*	–0.19	–					
(17) CFPQ restriction for health	–0.27*	0.22	0.28*	–0.04	0.19	–0.05	–0.15	–0.07	0.35**	0.05	–0.21	0.40**	0.32*	0.21	0.03	0.37**	–				
(18) CFPQ restriction for weight control	–0.17	–0.09	–0.04	0.22	–0.10	–0.22	–0.09	–0.14	0.17	0.05	–0.12	0.29*	0.27*	0.17	–0.05	0.29*	0.46**	–			
(19) CFPQ teaching about nutrition	0.04	0.20	0.23	–0.05	0.02	–0.08	–0.15	–0.21	–0.03	0.43**	0.04	0.08	0.48**	0.27*	0.29*	0.17	0.37**	0.25	–		
(20) FMGQ shared family food	–0.05	–0.11	0.07	–0.14	0.19	0.10	–0.10	–0.14	–0.01	–0.14	–0.16	0.06	–0.33*	–0.15	–0.22	0.06	–0.10	0.10	–0.11	–	
(21) FMGQ stress/conflict avoidance	–0.01	0.01	0.23	–0.13	0.12	0.25	–0.09	0.27*	0.04	–0.13	0.10	–0.03	0.08	0.02	–0.02	–0.03	–0.08	0.04	–0.04	0.26*	–
(22) FMGQ family involvement at mealtimes	–0.16	0.15	0.31*	0.03	0.18	0.14	–0.03	–0.18	0.15	0.01	0.17	0.19	0.55**	0.26*	0.21	–0.04	0.29*	0.27*	0.18	–0.04	0.28*

*p < 0.05, **p < 0.01.

EAS-T, Emotionality Activity Sociability–Temperament subscale (88); CFPQ, Comprehensive Feeding Practices Questionnaire (86); FMGQ, Family Mealtime Goals questionnaire (31).

**TABLE 8:** Correlations between vegetable consumption and parent and child measures, Polish sample (*N* = 122).

Variables	1	2	3	4	5	6
(1) Portions of veg per day	–					
(2) Childs age	–0.15	–				
(3) Food fussiness	–0.37**	0.29**	–			
(4) FMGQ shared family food	0.04	–0.03	–0.16	–		
(5) FMGQ stress/conflict avoidance	–0.02	0.02	0.00	0.30**	–	
(6) FMGQ family involvement at mealtimes	0.16	0.12	–0.09	0.24**	0.35**	–

*p < 0.05, **p < 0.01.

FMGQ, Family Mealtime Goals questionnaire (31).

#### Child food fussiness and temperament

Table 9 presents the mean food fussiness scores for the children in each country. There was no significant difference in food fussiness levels across countries [*F*(2,405) = 2.83, *p* = 0.06, η*^2^* = 0.01].

**TABLE 9:** Food fussiness (CEBQ:FF) scores for the children in the UK, Italy, and Poland.

	Whole sample	UK	Italy	Poland
*N*	408	225	61	122
	*M (SD)*	*M (SD)*	*M (SD)*	*M (SD)*
Food fussiness	3.07(0.86)	3.15 (0.78)	2.86 (1.09)	3.03 (0.85)

Table 10 presents the mean scores for the components of the Temperament subscale of the Emotionality Activity Sociability questionnaire (EAS-T) for children in Italy and the UK (this measure was not collected in Poland). The Italian children were rated more highly than the British children on both ‘emotionality’ [*t*(134.06) = –2.43, *p* = 0.02] and ‘sociability’ [*t*(90.17) = –5.15, *p* < 0.001].

**TABLE 10:** Emotionality activity sociability–temperament subscale (EAS-T) component scores.

	UK	Italy
*N*	*169*	*61*
Temperament component	*M (SD)*	*M*
Activity	4.08 (0.80)	4.11 (0.60)
Emotionality	2.62 (0.93)	2.90 (0.73)
Shyness	2.61 (0.75)	2.61 (0.75)
Sociability	3.04 (0.46)	3.45 (0.56)

To explore whether the individual child factors were related to vegetable intake, correlational analyses were conducted between intake and both food fussiness and the individual dimensions of child temperament (refer to Table 5). For the group as a whole, food fussiness was negatively correlated with reported vegetable intake [*r*(408) = –0.28, *p* < 0.001]; no other relationships were found (all *p*s > 0.05).

To investigate whether the individual child factors associated with children’s vegetable consumption differ between countries, analyses were conducted for each country separately (refer to Tables 6–8). Food fussiness was negatively correlated with vegetable intake in the UK [*r*(225) = –0.29, *p* < 0.001], Italy [*r*(61) = –0.27, *p* = 0.04], and Poland [*r*(122) = –0.37, *p* < 0.001]. In addition, vegetable intake was negatively related to the ‘emotionality’ [*r*(61) = –0.26, *p* = 0.05] and ‘shyness’ [*r*(61) = –0.36, *p* = 0.004] components of the EAS-T questionnaire for the Italian sample.

### Predictors of child vegetable intake and how these differed between countries

To investigate the relative and independent contributions of child, parent, and environmental measures in predicting children’s vegetable intake, we conducted a separate multiple regression analysis for each country. Each of the variables identified in the previous set of analyses as significantly associated with intake (*p* < 0.05) for a given country was included in an enter method multiple linear regression for that population. The data met the required assumptions for collinearity in each case, with no multicollinearity between the included predictors.

For the UK sample, the parent feeding style measures of CFPQ ‘environment,’ CFPQ ‘food as a reward,’ and FMG ‘family involvement’ were entered as predictors, as were child food fussiness and the responding parent’s level of education. The regression model significantly predicted vegetable intake [*R*^2^ = 0.13, *R*^2^ change = 0.13, *F*(5,162) = 4.69, *p* < 0.001], explaining 10% of the variance in consumption. Only child food fussiness (*B* = –0.55, *p* = 0.002) and FMG ‘family involvement’ (*B* = 0.35, *p* = 0.049) made significant unique contributions to the model.

For the Polish sample, child food fussiness was the only significant correlate of vegetable intake and the only factor to be entered into the model. The model was significant [*R*^2^ = 0.14, *R*^2^ change = 0.14, *F*(1,120) = 19.43, *p* < 0.001], explaining 13% of the variance in vegetable intake (food fussiness: *B* = –0.89, *p* < 0.001).

For the Italian sample, the parent feeding style factors of CFPQ ‘emotion regulation,’ CFPQ ‘food as a reward,’ and CFPQ ‘food restriction on health’ were entered into the model, as were the child factors of EAS-T ‘emotionality,’ EAS-T ‘shyness,’ and child food fussiness. The model significantly predicted vegetable intake [*R*^2^ = 0.26, *R*^2^ change = 0.26, *F*(6,54) = 3.13, *p* = 0.01], explaining 18% of the variance. However, only ‘shyness’ made a significant unique contribution to the model (*B* = –0.48, *p* = 0.01).

While the multiple regression findings are informative, they do not directly compare the extent to which variables predict vegetable intake between countries. To explore potential differences in the role of the predictors across countries, we adopted two different analytic approaches. First, we tested the predictor by country interactions for variables that were measured across groups. Second, we compared model fits, model structures, and beta coefficients between individual regression models that included shared predictors. Given that neither ‘attendance at day care’ nor ‘responding parent’s education’ was a significant predictor in the regressions for individual populations, we report the results of models excluding these sociodemographic variables. Their inclusion did not alter the results other than rendering one model for Italy non-significant (*p* > 0.05), likely because of the small sample size relative to the number of predictors entered.

To implement the first approach, we conducted a hierarchical regression model analysis to predict vegetable intake that included main and interaction effects. In the first block of predictors, the three countries were dummy-coded to allow for comparison between countries, with Poland as the reference group. This model significantly predicted vegetable intake [*R*^2^ = 0.06, *R*^2^ change = 0.07, *F*(2,405) = 14.52, *p* < 0.001]. In the second block, the shared predictors of interest were added, including age of child, food fussiness, and the FMG dimensions ‘shared family mealtimes,’ ‘stress/conflict avoidance,’ and ‘family involvement.’ The model was significant in predicting vegetable intake [*R*^2^ = 0.16, *R*^2^ change = 0.1, *F*(5,400) = 9.99, *p* < 0.001]. The third block included the interaction terms between the country variables and the shared predictors. Adding this block did not improve the model fit [*R*^2^ = 0.16, *R*^2^ change = 0.02, *F*(10,390) = 1.07, *p* = 0.39]. The model including the first and second blocks of predictors was therefore examined for significant beta coefficients. Vegetable intake in Poland was significantly greater than in the UK (*B* = –0.56, *p* = 0.003) and Italy (*B* = –1.4, *p* < 0.001). An alternative model with the UK as the reference group showed that vegetable intake was also higher in the UK than in Italy (*B* = –0.84, *p* = 0.001). The other significant predictors included food fussiness (*B* = –0.519, *p* < 0.001) and the FMG dimension ‘family involvement’ (*B* = 0.252, *p* = 0.005). Changes in the dummy-coding scheme did not change the model results or beta coefficients of non-country predictors. This hierarchical regression analysis therefore replicated the results of the ANOVA comparing vegetable intake across countries and further identified food fussiness and ‘family involvement’ as predictors of vegetable intake in the combined sample. However, the lack of significant interaction effects in the model suggests that this analysis was not sensitive to between-country differences in the effects of predictors.

Our second approach was to conduct separate, enter method, regression analyses for each country that included the same set of shared predictors (age, food fussiness, and FMG dimensions). Fisher’s *Z* tests were conducted to compare the *R* values of the regression for each country to assess the fit of the set of predictors in each case. The regression model was significant for the UK sample [*R*^2^ = 0.11, *R*^2^ change = 0.11, *F*(5,219) = 5.24, *p* < 0.001] and for the Polish sample [*R*^2^ = 0.17, *R*^2^ change = 0.17, *F*(5,116) = 4.62, *p* = 0.001] but not for the Italian sample [*R*^2^ = 0.16, *R*^2^ change = 0.16, *F*(5,55) = 2.08, *p* = 0.08]. The comparisons of model fit revealed no significant difference between the fit for the UK and Poland [*Z* = 0.815, *p* = 0.42], suggesting that the same predictor set worked well for the two countries. Food fussiness was a significant negative predictor of vegetable intake in both countries (UK: *B* = –0.5, *p* < 0.001; Poland: *B* = –0.71, *p* < 0.001). FMG ‘family involvement’ was also a significant positive predictor for the UK sample (*B* = 0.29, *p* = 0.02). We further compared the structure of the UK and Polish models by applying the model derived from the Polish sample to the UK dataset and comparing the crossed *R*^2^ with the direct *R*^2^. The model structure was not significantly different [*Z* = *0.51, p* = 0.610]. The regression weights for food fussiness did not differ between the two countries [*Z* = 0.948, *p* = 0.343], suggesting that the magnitude of this variable’s relationship with vegetable intake was similar in the two groups. Overall, these results corroborate those of the previous regression analyses. Because the model for the Italian sample was not significant, beta coefficients and model fit comparisons involving this group were not conducted.

Finally, given that a larger set of potential explanatory variables was collected in the UK and Italy, the above analysis was repeated using the common predictor set for these two countries. Separate enter method multiple regression models were used for the UK and Italy including the shared predictors of interest (age, food fussiness, FMG dimensions, CFPQ dimensions, and EAS-T dimensions). The model was significant for both the UK sample [*R*^2^ = 0.23, *R*^2^ change = 0.23, *F*(21,143) = 2.01, *p* = 0.009] and the Italian sample [*R*^2^ = 0.51, *R*^2^ change = 0.51, *F*(21, 39) = 1.91 *p* = *0.04*]. The Fisher’s *Z* test comparing the models’ fit was significant (*Z* = 2.424, *p* = 0.03); the larger *R*^2^ value for the Italian model indicates that the predictor set worked better for the Italian sample, suggesting that some of the additional parent and child variables helped to predict vegetable intake for this group. Further testing suggested structural differences between the two models (*Z* = 3.399, *p* < 0.001), confirmed by the lack of overlap between the significant predictor variables in the models. For the Italian sample, EAS-T ‘shyness’ was the only variable to make a significant contribution to the prediction of vegetable intake (*B* = -0.61, *p* = 0.001), echoing the results of the initial regression analysis. For the UK sample, food fussiness (*B* = –0.5, *p* = 0.003), EAS-T ‘emotionality’ (*B* = 0.34, *p* = 0.02), and CFPQ ‘food as a reward’ (*B* = –0.44, *p* = 0.007) all significantly contributed to the prediction of vegetable intake, presenting both consistencies and inconsistencies with the earlier regression results.

In sum, these regression analyses highlight food fussiness as a shared negative predictor of vegetable intake in the UK and Polish samples. In addition, FMG ‘family involvement’ was a significant positive predictor of intake for the UK sample. CFPQ ‘food as a reward’ and EAS-T ‘emotionality’ were identified as negative predictors for the UK sample in some analyses, although the results were inconsistent. No predictors were shared between the Italian sample and other countries. In Italy, EAS-T ‘shyness’ was the sole significant predictor of vegetable intake.

## Discussion

This study sought to compare the levels of vegetable intake in children from three European countries, Italy, Poland, and the UK, to explore how these are related to parent, child, and environmental factors previously found to influence vegetable consumption and healthy eating behaviour, and to identify whether the predictive relationships between these factors and vegetable intake are the same or differ between populations.

Previous reports have highlighted that the majority of children across Europe and around the globe do not meet public health guidelines on recommended levels of vegetable intake (11). The results of the current study corroborate these claims but also confirm previous findings that levels of vegetable intake vary across countries. Consistent with some previous findings (17), the Polish children were reported to eat the most vegetables among our groups, at around three child-sized portions per day, which was significantly more than the intake of children in the UK and Italy. Furthermore, the Italian children ate significantly fewer portions of vegetables than the children in the UK, only one portion per day on average. The children in all three countries consumed significantly less than the gold standard of five portions of vegetables per day (13); however, this may not be of concern if we consider that ‘5 a day’ guidelines typically include fruits and vegetables. A recent cohort study on adult mortality (90) concluded that good health is optimally supported by a diet consisting of three portions of vegetables and two portions of fruits per day. We therefore additionally compared children’s intake levels against the advised three portions of vegetables per day and found that the children in both the UK and Italy fell significantly short of this level of intake.

The variety of different vegetables reported to be eaten by children was also explored. The children ate an average of 10 different vegetables during the 2-week assessment period, suggesting that the parents are doing their best to offer a varied diet and that the children are not restricting their intake to a limited range of foods. Variety of intake was very highly correlated with the total quantity of intake (portions per day), and we found the same pattern of between-country differences in the variety of children’s diets as in the number of portions consumed. The Polish children consumed the widest range of vegetables, followed by the children in the UK, and then by the children in Italy.

It is important to consider whether methodological artefacts or third variables might be responsible for the observed differences in vegetable intake between countries. In terms of the former, might the differences be due to parents’ reporting accuracy, for example? Parents may not always be aware of all the foods their child eats, particularly if the child regularly attends day care. Indeed, we found that children who attended day care were reported to have lower levels of vegetable intake than children always cared for at home, when data from all three groups were pooled. However, this relationship did not hold for any individual country, and day care attendance had no predictive value in any model of vegetable intake either for the sample overall or for any individual group. The observed between-country differences in vegetable consumption therefore cannot be straightforwardly attributed to parents’ lack of awareness of the foods their child is eating at day care.

We also considered whether the questionnaires used to assess children’s vegetable intake might have differed between countries in ways that could have impacted the validity of parents’ responses. The original questionnaire, which was developed in English, excluded potatoes, which UK public health guidelines do not count as a vegetable because of their high carbohydrate content (14). When the UK questionnaire was adapted for use in Italy and Poland, additional vegetables that are commonly eaten in each local context were added to the list, while some rarely encountered vegetables were removed. In both adaptations, potatoes were added as a very commonly eaten vegetable; parents’ responses confirmed that these were the second and third most commonly consumed vegetable in Italy and Poland, respectively. To allow consumption levels to be compared across countries, potatoes were removed from the computed vegetable intake scores. While potato is also very commonly eaten by children in the UK, we note the possibility that the between-group differences we observed might have been less stark had potato been included for all the groups. Nevertheless, this consideration does not detract from the finding of differences in children’s intake of vegetables other than potatoes.

The finding that the Italian children were reported to consume the fewest portions and to have the lowest dietary variety among the three groups involved in our study corroborates other recent evaluations of preschool children’s diets (41) and challenges the stereotype that this group is likely to be fed a traditional Mediterranean diet high in fruits and vegetables. This finding is clearly a cause for concern and presents a more pessimistic picture than studies conducted just a few years before the current study (11, 18). It is worth noting that in our study, the data collection took place over a shorter time frame in Italy than in the other countries and during the winter months. However, whilst this might explain the lower variety of vegetables in Italian children’s diets, it does not account for the lower quantity of intake in this group. Rather, the results support the view that the rising childhood obesity rates seen in Southern European countries (10, 91) may be linked to the widespread transition from a traditional Mediterranean diet to a less vegetable-rich (i.e., more Western) diet in these populations (92). The results also suggest that the need for effective initiatives to promote vegetable intake and the potential to benefit from these varies between countries, and that Mediterranean regions may require particular support in improving the quality of children’s diets.

The second key aim of this study was to identify the environmental, parenting, and individual child measures associated with children’s vegetable consumption levels and to establish whether the influencing variables are the same or different across populations. To this end, we collected several questionnaire measures designed to assess factors that have previously been shown to play a role in children’s healthy eating. We then conducted correlational analyses followed by linear regression to identify the variables that made a significant contribution to models of vegetable intake. Follow-up analyses allowed us to establish which predictors were common across the groups and which were unique to a specific population.

The analyses implicated individual child factors as significant drivers of the vegetable intake of the preschoolers in our study. The Food Fussiness subscale of the Child Eating Behaviour Questionnaire (87) was collected for all the participants, and food fussiness was found to be negatively correlated with both the quantity and the variety of vegetables consumed in all three countries. These findings corroborate previous reports that food fussiness is an important determinant of dietary quality during the preschool years (56–59). Food fussiness peaks between 18 and 58 months of age (57), exactly the age range of children in our study, and has been shown to impact children’s willingness to consume new and familiar foods including vegetables (56–59). Despite the differing levels of vegetable intake (and variety of intake) between the children in each country, food fussiness levels were similar across the groups, corroborating the universality of fussy eating behaviour in children of this age. While food fussiness was negatively associated with vegetable intake in all three groups, it was a unique predictor of vegetable intake in the UK and Poland, where the magnitude of its impact was similar. Food fussiness was not a significant unique predictor of intake in the Italian sample, which may reflect the lack of power in the regression analysis due to the smaller size of this group; the fact that the correlation coefficient for the Italian participants was very similar to the correlation coefficient for the UK sample supports this suggestion. Failure to detect food fussiness as a significant unique predictor of vegetable intake in the Italian sample might alternatively (or additionally) reflect the important role played by other individual child factors in predicting vegetable intake (e.g., child temperament) and the variance these measures shared with food fussiness.

The Temperament subscale of the Emotional Activity Scale (EAS-T) (88) was administered in both the UK and Italy, with differences between the two groups identified on several subscales. Compared to the children in the UK, Italian children were reported to show higher emotionality, reflecting the quality and intensity of their emotional reactions, and higher sociability, indicating the extent to which children seek out and are gratified by social reward. Previous research has suggested that children with more emotional temperaments are more likely to show food avoidant behaviours (66), and that lack of sociability or ‘shyness’ is associated with feeding difficulties and being less willing to try new foods (40, 67). It is, therefore, possible that the low level of vegetable consumption of the Italian children was due, in part, to the higher levels of emotionality in this group (although their greater sociability should, to some extent, counteract the negative effects of their emotionality). However, the unique sole predictor of vegetable intake for the children in Italy was the ‘shyness’ component of the EAS-T. The role played by this factor was specific to this group; no correlation was seen between ‘shyness’ and vegetable consumption in the UK sample. The ‘emotionality’ subcomponent of EAS-T was negatively correlated with intake in the Italian sample, but did not make a significant contribution to the model for this group; rather, it emerged as a significant negative predictor of vegetable intake in some, but not all, of the analyses involving the UK sample. It is interesting to consider why child temperament, in general, and shyness, in particular, should be related to vegetable intake in children in Italy. One possibility is that Italian parents are more sensitive to their child’s temperament and more responsive to this when making decisions about how they feed their child. If that is the case, parents of shy children may need particular encouragement to include a variety of vegetables in their child’s diet.

Our study also explored the role of parenting factors in predicting children’s vegetable intake. The assessment of parents’ goals for family mealtimes [*via* the Family Mealtime Goals questionnaire (31)] revealed a number of differences between the groups. British and Polish parents endorsed the goals of sharing food as a family, avoiding stress and conflict at mealtimes, and involving family members in meal preparation to a greater extent than the Italian parents. The reasons why Italian parents are less preoccupied with these mealtime goals deserve investigation. It is possible that the differences in parents’ goals reflect differing cultural expectations in each country; for example, it might be more acceptable for families to eat separately or for family members to not be involved in preparing meals in Italy. Alternatively, and perhaps more plausibly, sharing foods, involving family members in meal preparation, and lower levels of stress during mealtimes may be the norm in Italy and, as a result, less likely to be considered goals that parents are striving to achieve.

In our study, the endorsement of the Family Mealtime Goal of ‘family involvement’ was positively associated with children’s vegetable intake and predicted unique variance in intake in the UK sample, over and above children’s food fussiness. Items contributing to this component included: “I want the whole family to help out with mealtimes,” “I want to choose food that my child can help prepare,” and “I want to get my child involved with things like setting the table or clearing up” (31). The importance of this component suggests that in the UK, at least, interventions that encourage and support parents to involve children in the preparation of meals and in contributing to mealtime activities might prove effective in increasing children’s vegetable intake.

A second parenting measure administered in the UK and Italy explored parents’ feeding styles using the Comprehensive Feeding Practices Questionnaire (86). Again, differences were seen in parents’ feeding practices across countries. The parents in the UK reported modelling healthy eating behaviours for their child and using food to regulate their child’s emotional state to a greater extent than the Italian parents. Modelling is a powerful influence on children’s early learning about the eating environment (19, 93); positive role-modelling can shape healthy eating behaviours (94) and support children in accepting new foods (34). In contrast, using food to regulate a child’s emotions is thought to be maladaptive, creating a relationship between emotions and food that can lead to overeating (95), increased BMI, and obesity (96). Why British parents adopt both more positive practices (modelling healthy eating) and more maladaptive practices (using food to regulate the child’s emotions) than Italian parents is unclear. One possible explanation relates to the extent to which parents feel able to influence their child’s eating behaviour. British parents may take a more ‘active’ approach because they believe their intervention has the potential to influence their child’s food preferences, causing them to attempt to manipulate their child’s eating behaviour in both positive and negative ways. In contrast, Italian parents may place more weight on factors internal to the child as determinants of what their child will eat, leading them to take a less agentive role in shaping their child’s eating behaviour. This hypothesis aligns with the finding that child temperament, specifically child shyness, is the key predictor of vegetable intake in Italian children. Italian parents may, quite rightly, believe that what their child eats is primarily determined by their child’s disposition, and intervene less as a result.

Interestingly, the parental feeding practices that differed between groups were not among the factors that predicted children’s vegetable intake. The only component of the Comprehensive Feeding Practices Questionnaire that appeared to play a role was ‘use of food as a reward,’ which showed a negative relationship with vegetable consumption in both groups in which it was assessed and emerged as a significant predictor in one regression analysis involving UK children. Previous studies have shown that food rewards are counter-productive, serving only to increase the desirability of the food used as a reward (the ‘treat’) while decreasing liking of the food that must be eaten to receive the reward (often a healthy food such as a vegetable) (97). Although we cannot be certain that the same pattern would have been true of children in Poland (where the CFPQ was not administered), the results suggest that avoidance of this parenting style may be universally important in efforts to increase children’s vegetable intake.

Further investigation is clearly needed to ascertain the basis of the cross-cultural differences our study has identified in parents’ mealtime goals and feeding behaviours and to better understand how child temperament interacts with parents’ feeding style and mealtime goals to determine healthy eating. A mixed methods approach would be most revealing in this endeavour. Highly powered quantitative studies that allow for interactions between parent and child factors to be included in regression models would help to unpick the weighting of these in determining children’s dietary quality. At the same time, qualitative approaches are more likely to provide an in-depth understanding of the differences in parents’ experiences of and approaches to feeding their families in different parts of Europe (76).

Finally, we also collected information about a range of sociodemographic characteristics to explore whether the environmental variables that have previously been linked to dietary quality and vegetable consumption levels were predictive of intake in our samples. Several variables differed between countries in our study. Specifically, the children in the Italian sample were older than those in the UK and Polish samples, fewer Polish children attended day care than the British or Italian children, and the Polish parents were more educated than those in Italy and the UK. However, none of these factors, including child age, contributed significantly to models of vegetable intake. As was discussed earlier, day care attendance was negatively associated with vegetable intake for the group overall, but this factor was not significant in either overall or by country regression analyses. Parent education (a proxy for socioeconomic status) is often found to be highly predictive of vegetable consumption (23), and it would therefore be logical to attribute the higher vegetable intake of the Polish children in our study to the higher educational levels of the parents in this group. However, while the educational level of the responding parents was associated with vegetable intake in our study, this was only true for the UK sample, and educational level was not a useful addition to any model of intake. We acknowledge, however, that the socioeconomic profile of our samples (as indexed by their education level) does not mirror the distribution of income and education in the wider populations from which they were drawn. The majority of participants in each of our groups had been university-educated, and the observed effect of parent education on child vegetable consumption was driven by differences between the reports of parents with higher degrees vs. those with bachelor’s degrees. In sum, while children’s vegetable intake in this study was not dependent on the background demographic characteristics of their family environment, a greater influence of the family environment might be seen among a more socioeconomically diverse group.

## Limitations

An obvious limitation of this study is that only a subset of the parent and child measures collected in the UK and Italy was collected in Poland and that the two measures not collected in Poland (EAS-T and CFPQ) were optional for the participants in the UK, impacting on sample sizes across our analyses. Administration of the full set of questionnaires in Poland would have allowed us to draw stronger conclusions about the predictors of vegetable intake that this group shared with the UK and/or Italy and might have revealed factors that were uniquely associated with children’s vegetable intake in Poland. Given the higher level of vegetable intake in Poland, further studies to identify the predictors of vegetable intake in Polish children would be worthwhile, as they might inform efforts to increase intake in populations where children are eating less wholesome diets.

Measures of additional variables known to influence children’s eating behaviour might also have been collected, such as measures of parents’ own food preferences, dietary choices, and eating behaviours, along with information about the participants in family mealtimes and the nature of the meals provided, which are likely to differ between the countries in which we collected the data. As in all studies, methodological decisions had to be made to ensure that key variables were collected while ensuring that the length of the survey was acceptable to the parents. We also note that all the measurements were reported by the parents rather than directly observed by researchers. While the validity of parent reports is sometimes called into question, a recent study by our group suggests that parents’ reports are reliable in the context of children’s eating behaviours (98). Parents are the primary gatekeepers of the foods young children eat and are therefore likely to be the most accurate recorders of children’s diets.

Other considerations to keep in mind when interpreting our findings include whether our participants were representative of the populations from which they were drawn and whether their reports can be relied upon to draw inferences about the questions of interest. Self-selecting participants in research studies tend not to be drawn equally from all demographic groups, and our sample reflects this bias; a large proportion of the parents in our study were educated to graduate or postgraduate level and identified as White. As discussed above, some questionnaires were optional, and the self-selection of those who choose to complete voluntary components of a study can introduce further bias. In this study, we considered the value of using the data contributed by the participants to outweigh concerns about potential bias given the large number of parents who completed the optional measures. The specific topic of a research study can also encourage bias in sampling. In this study, parents might have chosen to take part because they were concerned about their child’s vegetable intake or because they considered their child to be a fussy eater; this is particularly likely given that a subsequent phase of the study involved an intervention to increase children’s vegetable intake. We note too that the participants in each country were recruited via different channels, depending on the approach each research team considered would best achieve the target sample size. Different approaches to reaching participants might have led to differences between the participant pools. For example, the Polish sample was primarily recruited by participation in the national *Szkołanawidelcu* (School on a Fork) project, which may have biased the sample toward parents who were particularly mindful of healthy eating. The findings might therefore have been different if we had been able to recruit participants who were fully representative of the populations from which they were drawn.

Finally, we acknowledge that causal relationships between variables cannot be claimed on the basis of the correlational approach taken in this study. Indeed, while the most obvious explanation of a predictive relationship is often assumed to be the correct one, there are often alternative accounts that should be tested before they are discounted. For example, one might assume that the negative relationship between child food fussiness and vegetable intake is explained by fussy children’s reluctance to eat vegetables. However, it might alternatively reflect parents’ avoidance of offering vegetables to children who are perceived to be fussy, perhaps in an attempt to avoid scenes at mealtimes. In the current study, data on the quantity of vegetables offered to children were not collected separately from measures of children’s intake, making it impossible to tease these accounts apart. We anticipate that further exploration of the directionality and causes of the relationships this study has identified would reveal fruitful new avenues to supporting greater vegetable intake.

## Conclusion

This study has revealed both similarities and differences between the vegetable intake of preschool children in different European countries and the factors that drive these differences. The children in Poland consumed more vegetables than the children in the UK, who in turn consumed more vegetables than the children in Italy. The latter two groups fell significantly short of the guidelines on daily intake.

In terms of the predictors of vegetable consumption, child food fussiness was a negative correlate of vegetable intake in all the groups and a significant unique predictor of intake in the UK and Poland. Whilst this finding might indicate that fussy children are refusing to eat the vegetables offered to them, it might also indicate that parents of fussy children (or of children who are perceived to be fussy) are providing them with fewer vegetables at mealtimes; indeed both may be true. It is, of course, natural for parents to cease offering a food that their child has rejected several times previously (99). Many parents cannot afford food waste, while others may wish to avoid the mealtimes scenes that can occur when a disliked food is provided. However, children cannot eat foods that are not made available to them, and the literature has shown repeated exposure to a vegetable to be a powerful tool for bringing about acceptance (100, 101). Parents of fussy children may therefore need particular encouragement to be resilient in the face of food rejection.

Other predictors were found in only one or two of the populations involved in this study. Child temperament was a unique negative predictor of vegetable intake in Italy, where child shyness was associated with lower levels of consumption. Child emotionality was also negatively related to vegetable intake in some analyses involving children in the UK. The same argument applies to these groups as for children high in food fussiness; parents of shy or more emotional children (in Italy and the UK, respectively) may particularly benefit from support with encouraging healthy eating.

In terms of parents’ goals and behaviours, the results revealed higher levels of vegetable consumption in the UK among the children of parents who hold the goal of family involvement in mealtime preparation, suggesting that encouraging this strategy might be beneficial in increasing vegetable intake. The results of the analyses involving children in the UK and Italy also corroborate previous claims that using food as a reward is negatively associated with vegetable intake (97), confirming that interventions should discourage parents from this feeding behaviour.

These results highlight differences in both the extent to which European preschoolers achieve recommended levels of vegetable intake and in factors that influence whether they do. These findings imply that interventions to improve the quality of children’s diets require adaptation for the country in which they are implemented based on an understanding of baseline dietary quality and the specific factors that support or hinder the acceptance of healthy foods in that population.

## Data availability statement

The raw data supporting the conclusions of this article will be made available by the authors, without undue reservation.

## Ethics statement

The studies involving human participants were reviewed and approved in the UK by the University of Reading Research Ethics Committee, approval received 19th March 2019 (2019-018-CHP); in Italy by the Ethics Committee of the University of Turin, 2nd May 2019 (approval no. 176852); and in Poland by the Research Ethics Committee at the Faculty of Psychology, University of Warsaw, 8th April 2020. The participants provided their written informed consent to participate in this study.

## Author contributions

NM carried out the data analysis and wrote the first draft of the article. CH-P led the research team in developing the research questions and drafted the final version of the manuscript. NM, KD, KH, DB, MC, GC, PM, DM, and CH-P collaborated on the design of the study. NM, KD, DB, MC, GC, KW, DP, and JB were involved in the data collection and processing. KD, KH, DB, MC, GC, PM, KW, DP, JB, and DM provided feedback on the draft of the manuscript. All authors contributed to the article and approved the submitted version.

## Footnotes

1
https://osf.io/qjsdp

2 The non-parametric correlation (Spearman’s rho) between the CFPQ dimension ‘food as a reward’ and vegetable intake did not reach significance in the UK sample [*r*_*s*_(168) = –0.13, *p* = 0.09]. The non-parametric correlations (Spearman’s rho) between vegetable intake and the CFPQ dimensions ‘emotion regulation’ [*r*_*s*_(61) = –0.24, *p* = 0.06] and ‘food restriction for health’ [*r*_*s*_(61) = –0.225, *p* = 0.08] did not reach significance in the Italian sample. These factors were nevertheless included in the final regression model to err on the side of caution when including potential predictors of vegetable intake.

## Appendix

Vegetables listed in vegetable food frequency questionnaires in the UK, Italy, and Poland.

UK: artichoke, asparagus, aubergine, beetroot, broccoli, broad bean, Brussels sprouts, butternut squash, cabbage, carrots, cauliflower, courgette, cucumber, green beans, lettuce, leeks, mushroom, parsnip, peas, peppers, spinach, sweet potato, sweet corn, and tomato.

Italy: artichoke, asparagus, aubergine, beetroot, broccoli, broad bean, Brussels sprouts, butternut squash, cabbage, carrots, cauliflower, courgette, cucumber, green beans, lettuce, leeks, peas, peppers, spinach, tomato, potatoes, fennel, chard, and little tomato.

Poland: artichoke, asparagus, aubergine, beetroot, broccoli, broad bean, Brussels sprouts, cabbage, carrots, cauliflower, courgette, cucumber, green beans, lettuce, leeks, mushroom, peas, peppers, spinach, sweet corn, tomato, pumpkin, parsley, turnip, radish, potatoes, and onion.
